# Evaluation of measurement properties of self-administered PROMs aimed at patients with non-specific shoulder pain and “activity limitations”: a systematic review

**DOI:** 10.1007/s11136-016-1277-7

**Published:** 2016-04-02

**Authors:** M. Thoomes-de Graaf, G. G. M. Scholten-Peeters, J. M. Schellingerhout, A. M. Bourne, R. Buchbinder, M. Koehorst, C. B. Terwee, A. P. Verhagen

**Affiliations:** 1 Research Group Diagnostics, Avans University of Applied Science, Hogeschoollaan 1, 4818 CR Breda, The Netherlands; 2 Department of General Practice, Erasmus Medical Centre, Wytemaweg 80, 3015 CN Rotterdam, The Netherlands; 3 Faculty of Behavioural and Movement Sciences, MOVE Research Institute Amsterdam, VU University Amsterdam, Van der Boechorststraat 9, 1081 BT Amsterdam, The Netherlands; 4 Het Huisartsenteam De Keen, Voorsteven 88, 4871 DX Etten-Leur, The Netherlands; 5 Monash Department of Clinical Epidemiology, Cabrini Institute, Suite 41, 183 Wattletree Rd, Malvern, Melbourne, VIC 3144 Australia; 6 Department of Epidemiology and Preventive Medicine, The Alfred Centre, School of Public Health and Preventive Medicine, Monash University, 99 Commercial Road, Melbourne, VIC 3004 Australia; 7 SOMT Institute for Master Education in Musculoskeletal Therapies, Softwareweg 5, 3821 BN Amersfoort, The Netherlands; 8 Department of Epidemiology and Biostatistics and the EMGO Institute for Health and Care Research, VU University Medical Center, Van der Boechorststraat 7, 1081 BT Amsterdam, The Netherlands

**Keywords:** Shoulder pain, Disability, Questionnaire, Patient outcome assessment, Psychometrics, Systematic review

## Abstract

**Objective:**

To critically appraise and compare the measurement properties of self-administered patient-reported outcome measures (PROMs) focussing on the shoulder, assessing “activity limitations.”

**Study design:**

Systematic review. The study population had to consist of patients with shoulder pain. We excluded postoperative patients or patients with generic diseases. The methodological quality of the selected studies and the results of the measurement properties were critically appraised and rated using the COSMIN checklist.

**Results:**

Out of a total of 3427 unique hits, 31 articles, evaluating 7 different questionnaires, were included. The SPADI is the most frequently evaluated PROM and its measurement properties seem adequate apart from a lack of information regarding its measurement error and content validity.

**Conclusion:**

For English, Norwegian and Turkish users, we recommend to use the SPADI. Dutch users could use either the SDQ or the SST. In German, we recommend the DASH. In Tamil, Slovene, Spanish and the Danish languages, the evaluated PROMs were not yet of acceptable validity. None of these PROMs showed strong positive evidence for all measurement properties. We propose to develop a new shoulder PROM focused on activity limitations, taking new knowledge and techniques into account.

## Introduction

The International Classification of Functioning, Disability and Health (ICF) have described the widely accepted definition of functional health status in terms of “impairments,” “activity limitations,” and “participation restrictions” [1–3]. For patients with shoulder pain, one of the most important consequences in terms of their health is “activity limitations” [4]. As such, health-related patient-reported outcome measures (PROMs) that assess perceived “activity limitations” are useful in terms of assessing the physical impairment in patients with shoulder pain.

Several PROMs focusing on the shoulder have been developed to measure “activity limitations” in patients with shoulder pain. Examples of these include the Shoulder Disability Questionnaire (SDQ) [5] and the Shoulder Pain and Disability Index (SPADI) [6]. Furthermore, the disabilities of the arm, shoulder and hand questionnaire (DASH) is also often used for patients with shoulder pain [7]. There is a great variety in PROMs focusing on patients with shoulder pain. Some PROMs, such as the American Shoulder and Elbow Surgeon questionnaire (ASES), include a physical examination component, while others are completely self-administered. Other PROMs are specifically designed for a subgroup of patients, such as the wheelchair user’s shoulder pain index (WUSPI), which is specifically designed for wheelchair users.

Several systematic reviews have evaluated the measurement properties of shoulder-specific PROMS. A systematic review which included studies until 2002 found that none of the included 16 PROMs demonstrated satisfactory results for all measurement properties, but overall, the DASH received the best ratings [8]. Another review that assessed the measurement properties of four commonly used shoulder PROMs concluded that none of the questionnaires was superior or could be recommended over the other [9]. A recent review, specifically focused on patients with rotator cuff disorders (RCD), evaluated 12 PROMs and concluded that the included questionnaires showed acceptable psychometric properties for individuals with RCD [10]. Several other reviews have summarized the characteristics and measurement properties of a limited number of PROMs, but these reviews did not assess the methodological quality of the included studies and consequently their conclusions have several limitations [11–13].

Despite the fact that several reviews have been performed, we feel there is a need for a more specific and focused research question. If a research question is broad, it can be difficult to reach conclusions applicable to any single population. For example, a specific description of the patient population is important as it can influence the possibility to reach conclusions [14].

All of the above reviews included studies with mixed populations as well, such as upper extremity disorders. Their recommendations, about PROMs that can be used for patients with shoulder pain explicitly, are partly based on mixed populations, such as patients with solely hand or elbow pain (without shoulder pain). We feel that results of research on psychometric properties of shoulder PROMs should be based on data from patients with shoulder pain only, or should be presented separately. Study populations often consist of patients with “nonspecific” shoulder pain (including rotator cuff disease, frozen shoulder, etc.), but can also include patients with serious pathology (e.g., malignancy, infection and fracture), specific diseases (e.g., rheumatoid arthritis) or postsurgery patients. Especially if responsiveness is assessed, this can have consequences on the results. Therefore, we prefer to include only questionnaires assessing shoulder-related disability in patients with non-specific shoulder pain with or without conservative treatment.

Furthermore, these reviews presented their results per PROM and not per language; however, due to differences in cultural context, a translation of the original version does not guarantee similar psychometric properties [15, 16]. Therefore, the psychometric qualities of translated PROMs should also be evaluated, before they can be used in daily practice or research.

Recently, a new instrument known as the COSMIN checklist has been developed to evaluate the methodological quality of studies investigating the measurement properties of PROMs [17]. This checklist showed a high level of agreement between raters [17, 18]. Since its development, several systematic reviews examined the measurement properties of various PROMS by means of the COSMIN checklist [19–22].

Therefore, the aim of this study was to critically appraise and compare the measurement properties of both the original versions and the translated versions of self-administered PROMs focusing on the shoulder assessing “activity limitations” for patients with nonspecific shoulder pain, using the COSMIN checklist.

## Methods

### Selection criteria

We included publications concerning the development or validation/evaluation of measurement properties of an original or translated version of a self-administered PROM focussing on the shoulder and assessing “activity limitations”. Included patients should have nonspecific shoulder pain as a main complaint. As the definition of adhesive capsulitis, subacromial impingement syndrome and RCD is still unclear and there are no generally accepted criteria yet [23], we consider these pathologies as nonspecific shoulder pain and not as a specific subgroup. Studies including patients with serious pathology (e.g., malignancy, infection and fracture), specific diseases (e.g., rheumatoid arthritis) or where surgery was applied were excluded, as well as studies that did not report their results separately for patients with shoulder pain. Questionnaires including physical examination (e.g., ASES) were excluded, as well as questionnaires specifically designed for specific subgroups, such as RCD [e.g., Western Ontorio Rotator Cuff Index (WORC)], instability [e.g., Western Ontorio Shoulder Instability Index (WOSI)], athletes (e.g., Athletic shoulder outcome rating scale) or wheelchair users (e.g., WUSPI). We explicitly did not exclude studies in which patients with RCD, instability, etc., were used, but we chose to exclude all PROMs that were explicitly designed for a specific subgroup of shoulder complaints, as proposed by their developers.

No language restrictions were applied. Abstracts for which full reports were not available were excluded.

### Literature search

Electronic searches included MEDLINE, EMBASE, CINAHL and Cochrane from inception to August 2014. Eligible studies were identified using MeSH (Medline), Thesaurus (EMBASE, CINAHL) and free text words also including specific names of identified PROMs. We used the highly sensitive and precisely published search filter [24] for PubMed searches and used it to build the subsequent search strategies. We have added the MEDLINE search in the “Appendix,” the specific search strings for EMBASE, CINAHL and Cochrane are available from the authors on request. Manual searches of review bibliographies and reference lists of primary studies were also undertaken to search for possible studies not captured by the electronic searches.

A research librarian, together with a review author (MTG) performed the electronic search. Two review authors (MTG, GSP) independently selected the studies to be included by first screening the title and abstract and later assessing the full text papers for eligibility. Disagreements were solved by discussion or through arbitration by a third review author (AV). We listed the excluded studies and their bibliographic details with the reason for exclusion.

### Methodological quality

#### Quality assessment

Two reviewer authors (MTG and either JS, AB, MK or CT) independently performed the assessment of methodological quality, using the COSMIN checklist [17]. Disagreements were solved by discussion or by a third review author (AV). The checklist contains nine boxes, with standards for good methodological quality of studies on nine different measurement properties [17]. The appropriate boxes were selected per study and each item within this box scored on a 4-point rating scale: “poor,” “fair,” “good” or “excellent” [25]. An overall score for the methodological quality of a study was determined by taking the lowest rate of any items of the box per measurement property. An intraclass coefficient (ICC) was calculated to assess the immediate agreement between both raters on the overall score per box, and an ICC higher than 0.70 was considered good [26, 27].

#### Measurement properties

The measurement properties are divided into three domains: reliability, validity and responsiveness. Information on interpretability and feasibility were also extracted from the studies [17].

#### Interpretability

Interpretability is defined as: “the degree to which one can assign qualitative meaning-that is, clinical or commonly understood connotations- to an instrument’s quantitative scores or changes in scores” [28]. Information about clinically meaningful differences in scores between subgroups, floor and ceiling effects and the minimal important change (MIC) should be provided [17].

#### Reliability

Reliability is defined as: “the extent to which scores for patients who have not changed, are the same for repeated measurement under several conditions.” [28].

The reliability domain contains three measurement properties: internal consistency, reliability and measurement error [28]. Internal consistency is “the degree of the interrelatedness among the items” of the questionnaire [28] and is measured by Cronbach’s alpha or Kuder-Richardson Formula 20 or by using IRT methods [17, 27]. Reliability is “the proportion of the total variance in the measurements which is because of ‘true’ differences among patients” [28] and is reflected by the Intraclass Correlation Coefficient (ICC) or Cohen’s Kappa [17, 27]. The measurement error is “the systematic and random error of a patient’s score that is not attributed to true changes in the construct to be measured” [28]. This can be expressed by the standard error of measurement (SEM), the smallest detectable change (SDC) or the limits of agreement (LoA) [17, 27].

#### Validity

Validity is defined as: “the degree to which an instrument measures the construct(s) it purports to measure” [28]. The validity domain also contains three measurement properties: content validity, criterion validity and construct validity [28]. Content validity is “the degree to which the content of an instrument is an adequate reflection of the construct to be measured” and includes face validity [28]. The definition of face validity is “the degree to which (the items of) an instrument indeed looks as though they are an adequate reflection of the construct to be measured” [28]. In assessing this, it is important to consider whether all items are relevant to the originally described construct [17]. Criterion validity is “the degree to which the scores of an instrument are an adequate reflection of a ‘gold standard’” [28]. As PROMs do not have a “gold standard,” criterion validity is not appropriate [17]. Construct validity consists of three items:

1. Structural validity is “the degree to which the scores of an instrument are an adequate reflection of the dimensionality of the construct to be measured” [28]. Factor analysis should be used to determine or confirm existing subscales, which are subsequently used in the hypotheses that are being tested [28].
2. Hypotheses testing is “the degree to which the scores of an instrument are consistent with hypotheses (for instance with regard to internal relationships, relationships to scores of other instruments or differences between relevant groups. Based on the assumption that the instrument validly measures the construct to be measured)” [28].
3. Cross-cultural validity is “the degree to which the performance of the items on a translated or culturally adapted instrument is an adequate reflection of the performance of the items of the original version of the instrument” [28].

#### Responsiveness

Responsiveness is defined as: “the ability of an instrument to detect changes over time in the construct to be measured” [28]. Responsiveness is considered to be similar to validity; however, while validity refers to the validity of a single score, responsiveness refers to the validity of a change score [17].

### Data extraction

Two review authors independently performed data extraction (MTG and either JS, AB, MK or CB). Disagreements were resolved by discussion or by a third review author (AV). Descriptive data extracted included the characteristics of the study population (e.g., age, gender, type of shoulder pain, language); general characteristics of the instruments (e.g., construct, subscales, number of items); whether the PROM was an original version or a translated version of the questionnaire and feasibility. Although feasibility is not captured within the COSMIN checklist, the practical use of a questionnaire is important to determine usefulness in clinical practice. Feasibility includes the time needed to complete the questionnaire, its comprehensibility and whether or not it is generally accepted in clinical practice.

Besides, result of the measurement properties and of the interpretability was extracted. Only studies that were ranked as being of fair to excellent methodology were rated on their measurement properties, as studies of poor methodology are of limited value [19, 20].

To rate the results of measurement properties, generally accepted criteria were used [27].

### Analysis

To determine the overall quality of the measurement properties of the different questionnaires we combined the different studies per PROM (for each language) by combining their results (ratings), adjusted for the methodological quality (fair, good or excellent) and the consistency of their results. The overall rating for a measurement property was recorded as “positive,” “indeterminate” or “negative.” Furthermore, we assessed a level of evidence (strong, moderate, limited, conflicting, unknown) using the COSMIN checklist in a similar manner to that proposed by the Cochrane Review Group (see Table 1) [29].

**Table 1:** Levels of evidence for the overall quality of the measurement property

Level	Rating^a^	Criteria^b^
Strong	+++ OR − − −	Consistent findings among multiple studies of good/excellent methodological quality
Moderate	++ OR −	Consistent finding among multiple studies of fair studies or in one study of good methodological quality
Limited	+ OR −	One study of fair methodological quality
Conflicting	+/−	Conflicting findings
Unknown	?	Only studies of poor methodological quality
No evidence	0	No studies available

^a^Rating is based on Table 1 per study, where + refers to a positive result and − for a negative result

^b^The criteria of methodological quality are based on the COSMIN checklist

We made recommendations concerning the use of a certain PROM per language, based upon the best evidence synthesis. Ideally, a PROM should have strong positive evidence on all measurement properties; however, if there was moderate evidence, a recommendation was still made. In case multiple PROMs showed similar ratings in a specific language, both were presented. If there were no studies with at least fair methodology, no recommendations were made and if there was only limited evidence, caution was advised.

## Results

**Fig. 1:**
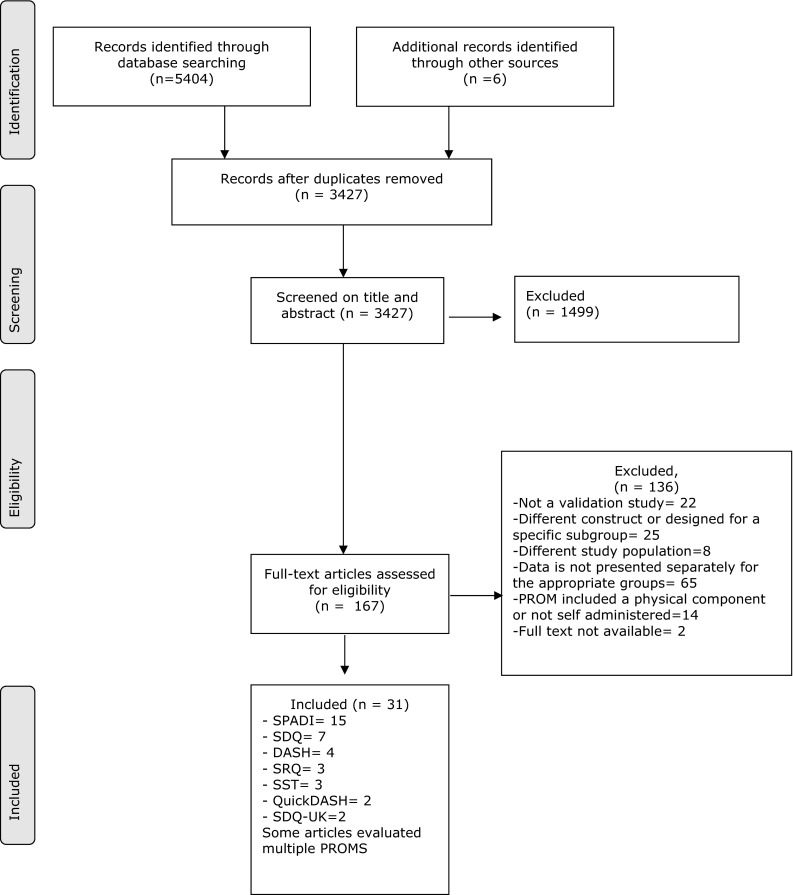
Inclusion

The search strategy resulted in a total of 3421 hits. Of these, 161 articles were selected based on their title and abstract. Reference checking resulted in 6 additional studies. Evaluation of the full text articles resulted in exclusion of 136 articles. Finally, 31 articles, evaluating 7 different questionnaires, were included (see Fig. 1).

The characteristics of the included studies are described in Table 2. For some articles, fewer boxes were scored than described by their original authors, as they did not present these results for our target population separately. The agreement between both raters on the methodological overall quality per box was good [ICC two way random agreement = 0.88 (95 % CI 0.818–0.915]. There was no need to discuss disagreement with the third review author. All original versions were developed in English, except the SDQ, which was originally developed in Dutch. The originally described construct and examples of questions of each PROM are described in Table 3. The methodological quality of the studies is presented in Table 4 for each PROM for each measurement property. The main categories with poor methodology were internal consistency, reliability and cross-cultural validity. The comparator instruments that were used for construct hypothesis testing (except studies of poor methodology) are presented in Table 5. The best evidence synthesis of results per language (per PROM) and their accompanying level of evidence are presented in Table 6.

**Table 2:** Characteristics of the included studies

Study	Country	PROMs	Setting	Population
English				
Beaton et al. [44]	Canada/USA	DASH	Hospital	Mixed types of shoulder pain Mean age 53, 43 % male^a^
Cloke et al. [63]	UK	SPADI	Shoulder clinic	Subacromial impingement Mean age 55, 44 % male
Croft et al. [54]	UK	SDQ-UK	GP	Shoulder pain Community mean age 65, 28 % male; General practice attendees mean age 51, 48 % male
Fan et al. [64]	USA	QuickDASH	Working population	Shoulder pain Mean age 40, 52 % male^a^
Godfrey et al. [53]	USA	SST	Hospital	Rotator cuff disease Mean age 42, 67 % male
Hill et al. [34]	Australia	SPADI	General population	Shoulder pain or stiffness Mean age 56, 41 % male
L’Insalata et al. [47]	USA	SRQ	Hospital	Mixed types of shoulder pain Mean age 40, 73 % male
MacDermid et al. [39]	Canada	SPADI	General population	Shoulder pain Mean age 44, 49 % male
Mintken et al. [52]	USA	QuickDASH	Physiotherapy	Shoulder pain Stable patients mean age 44, 59 % male; Improved patients mean age 39, 66 % male
Paul et al. [31]	UK	SDQ SDQ-UK SPADI SRQ	Shoulder clinic	Shoulder pain Mean age 54, 50 % male
Roach et al. [6]	USA	SPADI	GP	Shoulder pain Mean age 58, 100 % male
Staples et al. [40]	Australia	SPADI DASH	Physiotherapy	Adhesive capsulitis Mean age 56, 25 % male
Tashjian et al. [51]	USA	SST	GP	Rotator cuff disease Mean age 51, 48 % male
Dutch				
van der Heiden et al. [5]	Netherlands	SDQ	Rehabilitation clinic	Shoulder pain and stiffness Mean age 51, 49 % male
van Kampen et al. [50]	Netherlands	SST	Hospital	Shoulder pain Mean age 39, 72 % male
Vermeulen et al. [48]	Netherlands	SRQ	Hospital	Mixed types of shoulder pain Mean age 52, 23 % male
Norwegian				
Ekeberg et al. [37]	Norway	SPADI	GP	Rotator cuff disease Mean age 51, 34 % male
Ekeberg et al. [33]	Norway	SPADI	GP	Rotator cuff disease Mean age 51, 37 % male
Haldorsen et al. [45]	Norway	DASH	Outpatient clinic	Shoulder impingement Mean age 53, 52 % male
Tveita et al. [36]	Norway	SPADI	Hospital	Adhesive capsulitis Not reported
Tveita et al. [35]	Norway	SPADI	Hospital	Adhesive capsulitis Mean age 52, 42 % male
Turkish				
Bicer et al. [38]	Turkey	SPADI	Rehabilitation clinic	Shoulder pain Mean age 53, 0 % male
Dogu et al. [30]	Turkey	SDQ SPADI	Physiotherapy	Shoulder impingement Mean age 56, 33 % male
Ozsahin et al. [42]	Turkey	SDQ	Shoulder clinic	Shoulder pain Mean age 51, 25 % male
German				
Offenbacher et al. [65]	Germany	DASH	Hospital	Shoulder pain Mean age 59, 27 % male
Danish				
Christiansen et al. [32]	Denmark	SPADI	Hospital	Shoulder pain Mean age 48, 46 % male
Spanish				
Alvarez-Nemegyei et al. [66]	Mexico	SDQ	Hospital	Subacromial impingement Mean age 55, 20 % male
Slovene				
Jamnik et al. [41]	Slovenia	SPADI	Rehabilitation clinic	Chronic shoulder complaints Mean age 56, 29 % male
Tamil				
Jeldi et al. [67]	India	SPADI	Physiotherapy	Shoulder pain or dysfunction Mean age 49, 48 % male

^a^Based on whole cohort, not separately reported for the section of interest

**Table 3:** Overview of PROMs used with their originally described construct and an example of questions used

PROM	Description of the construct by the original author (and the author of a study assessing content validity)	Example of used questions
SPADI	Pain and disability [6]	1. How severe is your pain when…. When lying on the involved side? 2. How much difficulty did you have…. washing your back?
SDQ	Functional status limitation [5] Pain-related disability [43]	1. My shoulder hurts when I lie on it: Y/N 2. My shoulder is painful when I open or close a door: Y/N
DASH	Symptoms and functional status focused on physical function. The items tap upper extremity-related symptoms and measure functional status at the level of disability. Disability is defined as “difficulty doing activities in any domain of life (the domains typical for one’s age-sex group) due to a health or physical problem” [7]	Please circle the number that best describes your physical ability in the past week. Did you have any difficulty: 1. Using your usual technique for your work? 2. Doing your usual work because of arm, shoulder or hand pain? No difficulty (1)—Unable (5)
SRQ	Symptoms and function [47]	The following questions refer to pain: 1. During the past month, how would you describe the usual pain in your shoulder during activities? Very severe (1)—None (5) The following questions refer to daily activities: 1. During the past month, how much difficulty have you had in each of the following activities due to your shoulder; putting on or removing a pullover sweater or shirt? Unable (1)—No difficulty (5)
SST	Functional limitations of the affected shoulder [49]	1. Can you reach the small of your back to tuck in your shirt with your hand? Y/N 2. Can you place your hand behind your head with the elbow straight out to the side? Y/N
QuickDASH	Physical function and symptoms in persons with any or multiple musculoskeletal disorders of the upper limb [58]	Please rate your ability to do the following activities in the last week by circling the number below the appropriate response 1. Open a tight or new jar 2. Do heavy household chores (e.g., wash walls, floors) No difficulty (1)—Unable (5)
SDQ-UK	Disability associated with shoulder symptoms [54]	1. Because of my shoulder, I move my arm or hand with some difficulty: Y/N 2. I do not bath myself completely because of my shoulder: Y/N

**Table 4:** Methodological quality of each study per measurement property

Study	Internal consistency	Reliability	Measurement error	Content validity	Structural validity	Hypotheses testing	Cross-cultural validity/*only a translation	Responsiveness
SPADI developed in English								
Bicer et al. [38]	Poor	Fair				Fair		
Christiansen et al. [32]	Poor	Poor	Poor			Fair	Poor	
Cloke et al. [63]		Poor				Poor		Poor
Dogu et al. [30]								Poor
Ekeberg et al. [37]	Poor	Good	Good			Fair		
Ekeberg et al. [33]								Good
Hill et al. [34]	Excellent				Good	Poor		
Jamnik et al. [41]	Poor	Poor			Poor	Fair	Fair*	Poor
Jeldi et al. [67]	Poor	Poor				Poor	Poor	
MacDermid et al. [39]	Fair				Fair	Fair		Poor
Paul et al. [31]						Fair		Fair
Roach et al. [6]	Poor	Poor			Poor	Poor		Poor
Staples et al. [40]						Fair		Fair
Tveita et al. [36]		Fair	Fair				Fair*	Poor
Tveita et al. [35]	Fair				Fair			
SDQ developed in Dutch								
Alvarez-Nemegyei et al. [66]	Poor	Poor					Poor	
Dogu et al. [30]								Poor
van der Heiden et al. [5]								Fair
Ozsahin et al. [42]	Poor	Fair				Poor	Poor*	
Paul et al. [31]						Fair		Fair
van der Windt et al. [4]								Good
de Winter et al. [43]	Poor			Excellent		Fair		
DASH developed in English								
Beaton et al. [44]						Fair		
Haldorsen et al. [45]	Poor	Fair	Fair			Fair		
Offenbacher et al. [65]	Poor	Poor				Fair	Poor*	
Staples et al. [40]						Fair		Fair
SRQ developed in English								
L’Insalata et al. [47]	Poor	Poor				Poor		
Paul et al. [31]						Fair		Fair
Vermeulen et al. [48]	Poor	Fair				Poor	Excellent*	
SST developed in English								
Godfrey et al. [53]						Poor		
van Kampen et al. [50]	Excellent	Fair	Fair		Excellent	Good	Fair*	
Tasjian et al. [51]								Poor
QuickDASH developed in English								
Fan et al. [64]						Poor		
Mintken et al. [52]		Poor	Poor					Fair
SDQ-UK developed in English								
Croft et al. [54]				Poor		Poor		
Paul et al. [31]						Fair		Fair

* only a translation

**Table 5:** Comparator instrument in case of hypothesis testing

Study	Comparator instruments and correlations
SPADI	
Bicer et al. [38]	Convergent: the Spearman correlation with the HAQ total score was 0.67 and 0.65 with VAS during AROM
Christiansen et al. [32]	Known groups: those currently working, despite their shoulder pain, were found to have significantly lower scores than those not working; the mean difference was −18.3 (95 % CI −29.4 to −7.2)
Ekeberg et al. [37]	Convergent: the Spearman correlation with the OSS total score was 0.57, −0.67 for the WORC total, −0.75 with WORC physical, −0.46 with WORC Sports, −0.55 with WORC Work and −0.69 with WORC Lifestyle Divergent: the Spearman correlation between the SPADI and the WORC emotions was −0.31
Jamnik et al. [41]	Known groups: participants who differed in the severity of the perceived disability self-rating (mild–moderate–severe) differed significantly in the SPADI score in the presumed order
MacDermid et al. [39]	Known groups: patients who had diagnosed shoulder problems and those on pain mediation reported significantly higher pain and disability scores. Convergent: convergent scales (Home management 0.59, Work −0.10, Physical dimension 0.51) of the SIP showed a moderate correlation, except the work scale Divergent: divergent (emotional) scales of the SIP showed low correlations (0.17–0.33)^a^
Paul et al. [31]	Convergent: the spearman correlation with other shoulder PROMs was: 0.57 for the SDQ-UK, 0.33 with the SDQ and 0.83 with the SRQ. The correlation with Difficulty VAS 0.62^a^
Staples et al. [40]	Convergent: the Pearson correlation with other shoulder PROMs was: 0.55 with the DASH and 0.65 with the Croft index. Correlations with generic PROMs were: 0.17 with PET, 0.60 with Pain and 0.55 with the HAQ
SDQ	
Paul et al. [31]	Convergent: the Spearman correlation with other shoulder PROMs was: 0.55 for the SDQ-UK, 0.33 with the SPADI and 0.43 with the SRQ. The correlation with Difficulty VAS 0.47^a^
de Winter et al. [43]	Known groups: significant differences in the SDQ scores (*p* < 0.001) were found for subgroups with different pain severity, ability to perform activities in daily life, mobility, muscle force, and levels of disability according to the physical therapists. Convergent: the Spearman correlation with severity of disability was 0.58, and degree of difficulty for the main functional limitation was 0.32^a^
DASH	
Beaton et al. [44]	Known groups: those currently working with their upper limb condition and able to continue doing so had significantly lower disability than those who were not able to work (26.8 vs. 50.7, *t* = −7.51, *p* < 0.001). Statistically significant differences were also found between those who were able to do all they want to do as opposed to those who were not able to do so (23.6 vs. 47.1, *t* = −5.81, *p* < 0.0001). Convergent: The Spearman correlation with the overall rating of the problem was 0.68, with the ability to function 0.85, with the ability to work 0.76, with Brigham symptoms 0.71 and 0.90 with Brigham symptoms. The Spearman correlation with another shoulder PROM 0.76 with the SPADI pain scale and 0.83 with the SPADI function scale^a^
Haldorsen et al. [45]	Convergent: the Pearson correlation with the SPADI was 0.75 and with the NPRS 0.58. The correlations with components of the SF-36 were: physical functioning −0.48, bodily pain −0.62, and physical component summary −0.59 Divergent: the Pearson correlation with the mental component summary score of the SF-36 was −0.17 and −0.35 with the social functioning scale of the SF-36
Offenbacher et al. [65]	Convergent: the Spearman correlation with the HAQ was 0.81, with the SF-36 physical functioning component −0.58, and with global impact 0.76^a^
Staples et al. [40]	Convergent: the Pearson correlation with other shoulder PROMs was: 0.55 with the SPADI and 0.65 with the Croft index. Correlations with generic PROMs were: 0.20 with PET and 0.54 with the HAQ^a^
SRQ	
Paul et al. [31]	Convergent: the spearman correlation with other shoulder PROMs was: 0.72 for the SDQ-UK, 0.83 with the SPADI and 0.43 with the SDQ. The correlation with Difficulty VAS 0.60^a^
SST	
Kampen van et al. [50]	Convergent: the Pearson correlation with other shoulder PROMs was: 0.74 with the OSS, 0.59 with the CM and 0.74 with the DASH. The correlation with the SF-36 subscale physical functioning was 0.56
SDQ-UK	
Paul et al. [31]	Convergent: the Spearman correlation with other shoulder PROMs was: 0.72 for the SRQ, 0.57 with the SPADI and 0.55 with the SDQ. The correlation with Difficulty VAS 0.41^a^

^a^ROM, pain alone and the EQ5D were considered to be inappropriate comparators and were therefore excluded in the rating process

**Table 6:** Best evidence synthesis

PROM	Internal consistency	Reliability	Measurement error	Content validity	Structural validity	Hypotheses testing	Cross-cultural validity	Responsiveness
English								
SPADI	+++	?	0	0	++	++	0	++
DASH	0	0	0	0	0	++	0	+
SDQ-UK	0	0	0	?	0	+	0	+
SRQ	?	?	0	0	0	+	0	+
SDQ-English	0	0	0	0	0	–	0	+
SST	0	0	0	0	0	?	0	?^d^
QuickDASH	0	0	0	0	0	?	0	0
Dutch								
SST-Dutch	+++	+	?^a^	0	+++	++	0	0
SDQ	?	0	0	?^b^	0	+	0	++
Quick DASH-Dutch	0	?	?	0	0	0	0	+
SRQ-Dutch	?	+	0	0	0	?	0	0
Norwegian								
SPADI-Norwegian	+	++	?^a^	0	–	+	0	++
DASH-Norwegian	?	+	?^a^	0	0	+	0	0
Turkish								
SPADI-Turkish	?	+	0	0	0	+	0	?
SDQ-Turkish	?	+	0	0	0	?	0	?
German								
DASH-German	?	?	0	0	0	+	0	0
Danish								
SPADI-Danish	?	?	?	0	0	?^c^	?	0
Spanish								
SDQ-Spanish (Mexican)	?	?	0	0	0	0	?	0
Slovene								
SPADI-Slovene	?	?	0	0	?	?^c^	0	?
Tamil								
SPADI-Tamil	?	?	0	0	0	?	?	0

^a^Despite fair/good methodology, the level of evidence could not be determined as the appropriate measurement properties were not provided

^b^Despite fair/good methodology, the level of evidence could not be determined as the originally described construct differed from the construct described in the current study

^c^Despite fair/good methodology, the level of evidence could not be determined as unclear, as they confirmed their hypothesis with known group validity, but did not assess whether the correlations with related constructs were higher than with unrelated constructs

^d^This study only evaluated the minimal clinical difference

Below we will describe the results per questionnaire.

### Shoulder pain and disability index (SPADI)

The SPADI was developed to measure pain and disability associated with shoulder pathology. It consists of 13 items, each scored on a 0–10 numeric rating scale, divided into two subscales: pain (5 items) and disability (8 items). The total score varies between 0 and 100 [6]. It takes approximately 2–3 min to complete [30, 31]. The SPADI is considered to be easy to understand by patients [31], and no floor or ceiling effects have been detected [32, 33].

#### Reliability

##### Internal consistency

There is strong positive evidence for internal consistency within the English SPADI (Cronbach Alpha = 0.85 for pain and 0.90 for disability) [34]. There is also limited positive evidence for the internal consistency of the Norwegian SPADI (Cronbach Alpha = 0.80 for pain and 0.87 for disability) [35]. However, there were inconsistent findings on the factor structure of the SPADI; therefore, these results should be interpreted with caution.

##### Reliability

Both the Norwegian and the Turkish versions showed moderate (ICC = 0.85–0.89) [36, 37] and limited positive evidence (ICC = 0.92) [38], respectively. Studies evaluating other language versions were rated as having poor methodology.

##### Measurement error

Two studies (both Norwegian) were rated as having at least “fair” methodology that evaluated measurement error, one study of fair methodology only reported an SDC (17 points), but no MIC was determined [36]. The other study reported an SDC of 19.7, and the Loa was between -20.9 and 18.5 [37]; the MIC, however, ranged between 15.0 and 31.1 depending on the methods used [33]; the authors therefore concluded that a change of approximately 20 points is necessary for patient perceived important change.

#### Validity

##### Content validity

There were no studies evaluating content validity.

##### Construct structural validity

There is moderate evidence that the English SPADI consists of two factors, pain and disability, and all factors are loaded accordingly as originally proposed by Roach [34]. In contrast, there is limited evidence that not all items are loaded on the original factor, but no explained variance was described [39]. Factor analysis of the Norwegian SPADI resulted in limited evidence that it consists of two factors but the original factor structure could not be confirmed, as not all items loaded as originally intended [35].

##### Construct hypothesis testing

In terms of construct hypothesis testing, moderate positive evidence was identified for the English SPADI [31, 39, 40]. There was limited positive evidence for the Turkish version [38] and the Norwegian version [37]. The evidence for the Danish SPADI [32] and the Slovenish version [41] was unclear, as they confirmed their hypothesis with known group validity, but did not assess whether the correlations with related constructs were higher than with unrelated constructs.

##### Construct cross-cultural validity

Only studies that were rated as being of poor methodology have been performed.

#### Responsiveness

There is moderate positive evidence for responsiveness of the English version (AUC ranging between 0.74 and 0.87) [31, 40] and the Norwegian version (AUC = 0.84 or 0.92 depending on the follow-up period) [33].

### Shoulder Disability Questionnaire (SDQ)

The SDQ is 16-item pain-related disability questionnaire that was originally developed in Dutch. Response options are “yes,” “no” or “not applicable,” resulting in a total score which ranges from 0 to 100, with a higher score indicating more severe disability [4]. It takes about 2 [30, 31] to 4 min to complete, and patients indicated the SDQ as (very) easy to complete [5, 30, 31]. One study assessed whether there were signs of floor or ceiling effects; however, they did not report the data needed to give a proper indication of it [5].

#### Reliability

##### Internal consistency

Only studies that were rated as being of poor methodology have been performed.

##### Reliability

There were no sound methodological studies evaluating reliability, except for the Turkish version, which showed limited positive evidence, with a Pearson correlation coefficient of 0.88 for the total score [42].

##### Measurement error

There were no studies evaluating the measurement error.

#### Validity

##### Content validity

The evidence regarding content validity of the original SDQ is indeterminate, as the questions are not aimed at the originally described construct (see Table 4).

##### Construct structural validity

There were no studies evaluating structural validity.

##### Construct hypothesis testing

There is limited positive evidence for the Dutch version [43] and limited negative evidence for the English version (as three out of the seven expected positive correlations measured were below 0.50) [31].

##### Construct cross-cultural validity

No studies specifically assessed cross-cultural validity.

#### Responsiveness

There is moderate positive evidence for the Dutch version (AUC = 0.84) [4] and limited positive evidence for the English version (AUC = 0.77) [31].

### Disability of arm, shoulder and hand (DASH)

The DASH is designed to measure symptoms and physical functioning in patients with pain in the arm, shoulder or hand. It consists of 30 items, and the response options for each item are presented as 5-point Likert scales. The total score ranges from 0 to 100 [7]. We did not find studies reporting any item on feasibility. No floor or ceiling effects were detected [44, 45].

#### Reliability

##### Internal consistency

Only studies that were rated as being of poor methodology have been performed.

##### Reliability

There is limited positive evidence for the Norwegian version (ICC = 0.89) [45].

##### Measurement error

The result of the only study with fair methodology evaluating measurement error is indeterminate, as they did not provide the MIC; the SDC, however, was 6.7 points for the Norwegian version [45].

#### Validity

##### Content validity

There were no studies evaluating content validity.

##### Construct structural validity

There were no studies evaluating structural validity.

##### Construct hypothesis testing

There is moderate positive evidence for construct hypothesis testing of the English version [40, 44] and limited positive evidence for the German [46] and Norwegian version [45].

##### Construct cross-cultural validity

No studies specifically assessed cross-cultural validity.

#### Responsiveness

There is limited positive evidence for the English version for responsiveness (AUC = 0.71–0.86 depending on the anchor used) [40].

### Shoulder Rating Questionnaire (SRQ)

The SRQ was developed to measure the severity of symptoms related to and the functional status of the shoulder. It covers seven domains including 21 items—the total score ranges between 17 and 100 [47]—takes about 4 [31] to 7 [48] minutes to complete and is moderately easy to complete according to patients [31]

#### Reliability

##### Internal consistency

Only studies that were rated as being of poor methodology have been performed.

##### Reliability

There was limited positive evidence for the reliability of the Dutch version (ICC = 0.85) [48].

##### Measurement error

There were no studies evaluating the measurement error.

#### Validity

##### Content validity

There were no studies evaluating content validity.

##### Construct structural validity

There were no studies evaluating structural validity.

##### Construct hypothesis testing

There was limited positive evidence for the English SRQ [31].

##### Construct cross-cultural validity

No studies specifically assessed cross-cultural validity.

#### Responsiveness

There was limited positive evidence for the responsiveness of the English SRQ (AUC = 0.85) [31].

### Simple shoulder test (SST)

The SST was developed to measure functional limitations in patients with shoulder dysfunction. It consists of 12 items, and the response options are dichotomous. The total score ranges between 0 and 12 [49]. We did not find studies reporting any item on feasibility.

No floor or ceiling effects were detected [50].

#### Reliability

##### Internal consistency

There was strong positive evidence for the Dutch SST with a Cronbach Alpha of 0.78 [50].

##### Reliability

There was limited positive evidence for the reliability of the Dutch SST (ICC = 0.92) [50].

##### Measurement error

The result of the only study with fair methodology evaluating measurement error is indeterminate, as they did not provide the MIC; the SDC, however, was 3.3 [50].

#### Validity

##### Content validity

There were no studies evaluating content validity.

##### Construct structural validity

There was strong evidence for the unidimensionality of the Dutch SST. Confirmatory factor analysis of a 1-factor model showed a moderate fit (CFI 0.94, TLI 0.93, RMSEA 0.07), and three items showed relatively low factor loadings [50].

##### Construct hypothesis testing

There is moderate positive evidence for construct hypothesis testing of the Dutch SST [50].

##### Construct cross-cultural validity

No studies specifically assessed cross-cultural validity.

#### Responsiveness

There were no studies judged as having a sound methodology evaluating the English version. One study on the English SST only calculated the minimal clinically important difference, but did not assess the responsiveness [51].

### QuickDASH

The QuickDASH is an 11-item questionnaire that addresses symptoms and physical function in people with disorders of the arm, shoulder or hand. It provides a summative percentage score, with 100 indicating the most disability [52]. We did not find studies reporting on feasibility. No floor or ceiling effects were detected [53].

#### Reliability

##### Internal consistency

There were no studies evaluating internal consistency.

##### Reliability

Only studies that were rated as being of poor methodology have been performed.

##### Measurement error

Only studies that were rated as being of poor methodology have been performed.

#### Validity

##### Content validity

There were no studies evaluating content validity.

##### Construct structural validity

There were no studies evaluating structural validity.

##### Construct hypothesis testing

Only studies that were rated as being of poor methodology have been performed.

##### Construct cross-cultural validity

No studies specifically assessed cross-cultural validity.

#### Responsiveness

There was limited positive evidence for responsiveness in the Dutch version (AUC = 0.82) [52].

### Shoulder Disability Questionnaire (SDQ-UK)

The SDQ-UK is a 22-item questionnaire [54]. The questionnaire contains some statements that people have used to describe themselves when they have trouble with their shoulder. Participants are asked to answer “yes” or “no” depending on whether they recognize the statement as applying to them, with a total score ranging between 0 and 100. It takes about 3 min to complete and patients describe it as easy to understand [31].

#### Reliability

##### Internal consistency

There were no studies evaluating internal consistency.

##### Reliability

There were no studies evaluating reliability.

##### Measurement error

There were no studies evaluating the measurement error.

#### Validity

##### Content validity

Only studies that were rated as being of poor methodology have been performed.

##### Construct structural validity

There were no studies evaluating structural validity.

##### Construct hypothesis testing

There was limited positive evidence for construct hypothesis testing [31].

##### Construct cross-cultural validity

No studies specifically assessed cross-cultural validity.

#### Responsiveness

There was limited positive evidence for the responsiveness (AUC = 0.77) [31].

### Recommended PROMS per language

#### English

All seven PROMs were available and assessed in English. For English users, we recommend using the English SPADI as it was rated best in the best evidence synthesis. It consists of two factors: There is strong positive evidence for the internal consistency and moderate evidence for construct hypothesis testing and the responsiveness.

#### Dutch

Four questionnaires were available and assessed in Dutch in this specific population. The SDQ was developed in Dutch, and the other three were developed in English. Both the SDQ and SST showed acceptable ratings in the best evidence synthesis. There was strong evidence for the reliability as well as for the construct validity for the Dutch SST. Strong positive evidence was found for the internal consistency and limited positive evidence for the reliability of the Dutch SST, and inconclusive evidence for the measurement error. The construct validity of the SST was strong, as there was strong evidence for the unidimensionality and moderate positive evidence for construct hypothesis testing.

There is limited positive evidence for construct hypothesis testing of the Dutch SDQ, and there is moderate positive evidence for responsiveness. We recommend choosing between either the SST or the SDQ depending on the purpose of its use.

#### Norwegian

Out of the two available instruments, the SPADI showed the best ratings. There is moderate positive evidence for the reliability and inconclusive evidence for the measurement error. There was limited evidence that the Norwegian SPADI did not follow the original factor structure and limited positive evidence for the internal consistency. There was limited positive evidence for construct hypothesis testing and moderate positive evidence for the responsiveness.

#### Turkish

In Turkish, both the SDQ and the SPADI were evaluated, and both only showed limited evidence; however, the SPADI also had limited evidence for construct hypothesis testing instead of only limited evidence for reliability. We therefore recommend using the SPADI, however, caution is advised.

#### German

We only found one study using a PROM in German when using our search criteria. There is limited positive evidence for the construct hypothesis of the German DASH. We recommend using the DASH in the German language; however, it is important to be aware of the lack of information available about this PROM in German.

#### Other languages

In Danish, Tamil and Slovene, the only instrument evaluated was the SPADI, in Spanish the only questionnaire assessed was the SDQ. For all four languages, we only found studies with poor methodology or information was missing regarding a measurement property. We could therefore not make a recommendation in these languages.

## Discussion

The SPADI has been the most frequently evaluated questionnaire in this review on patients with shoulder pain and its measurement properties seem adequate apart from a lack of information regarding its reliability, measurement error and content validity. For English users, we recommend its use, as this is the PROM with the best measurement properties.

For Norwegian users, the SPADI is recommended, as well for Turkish users, although for the latter caution is advised as the evidence is limited and information on some measurement properties is lacking. Dutch users could use either the SDQ or the SST, depending on the intended purpose. Germans could use the DASH, although caution is advised, as there is still a lack of information regarding many measurement properties.

In Danish, Spanish, Tamil and Slovene, the evaluated PROMs were not yet of acceptable validity. We found no studies concerning PROMs in other languages, which met our inclusion criteria.

### Comparison with the literature

One systematic review, assessing the methodological quality of measurement properties of shoulder PROMs, concluded that the DASH received the best ratings [8]. This is in contrast with our findings. A possible reason for this difference is the search period. Most studies reporting on the SPADI in our review were published after the search period (2002) of the previous review. Moreover, we excluded studies evaluating the DASH that did not report their results for shoulder pain patients separately.

Another recent review concluded that all of the included PROMs showed acceptable psychometric properties [10]. This study recommended PROMs that we excluded in our review [10]. The methodological quality of the studies included ranged from 33.3 to 95.9 %. No evidence synthesis was performed, and the psychometric properties per PROM were presented but without the methodological quality per study [10].

A review that evaluated the DASH, ASES, SPADI and SST only concluded that their measurement properties were acceptable and that none of the questionnaires was superior or could be recommended over the other. The quality of the individual studies ranged from 25 to 96 % [9]. This study presented the psychometric properties of all included studies, but did not use the methodological quality of the studies themselves in their conclusions about the psychometric properties of an instrument.

Our search strategy was designed to be highly sensitive rather than specific, resulting in a higher number of hits (3421) compared to other reviews [8–10, 12]. Two reviews did not describe their search strategy [11, 13], and two reviews also included studies that were not designed to validate a PROM [9, 10].

Most importantly, these reviews used an unspecified study population (e.g., including postoperative patients), included PROMs focused on a specific pathology (e.g., instability) and PROMs that included a physical component. We specified our study population and excluded studies that did not report their results for patients with shoulder pain separately. As a consequence, we excluded a high amount of studies that were focused on the DASH. Due to our strict selection criteria, we also excluded a number of well-known PROMs, due to our specific research question, such as the WOSI, a PROM that is designed specifically for patients with instability, or the ASES, which includes a physical component.

The major flaws we found with respect to the methodology are comparable with another study on measurement properties of neck pain and disability questionnaires [55]. For internal consistency, most studies did not measure the unidimensionality of the scale. The time interval and the sample size were the main problems within the reliability category, and sample size or performing a confirmatory analysis for cross-cultural validity.

### Strengths and limitations

We excluded two studies because we could not retrieve them as full text papers. One was written in Turkish. This could potentially have led to selection bias. However, the leading journals, and consequently the most important papers, are published in English.

We pooled our results by language rather than by country although we recognize that cultural differences may exist between countries. This means that for the English versions of PROMs, we pooled data from the UK, USA, Canada and Australia, hereby neglecting possible cultural differences. If countries are very close in location/culture/use of language and the text does not contain wording about education, health systems, brand names or IT, it is acceptable to use the same language version and to pool data from trials [56]. With respect to this, we assumed there are no insurmountable differences between the UK, USA, Canada and Australia. Moreover, our results did not show inconsistencies regarding measurement properties.

We excluded patients with generic and serious conditions (e.g., rheumatoid arthritis, fractures) and postoperative patients; therefore, our results cannot be extrapolated to these kinds of patients. The DASH is designed for patients with upper extremity disorders. Our conclusion on the DASH and its measurement properties are based on patients with shoulder pain only. Our results are therefore incomplete regarding the measurement properties of the DASH itself and cannot be extrapolated to other groups of patients on which the DASH can be used.

### Considerations regarding the results

We found that content validity of most PROMs is still unknown (a PROM should have evidence supporting its content validity, including evidence that patients and/or experts consider the content of the PROM relevant and comprehensive for the concept, population, and aim of the measurement application [57]), although content validity is often considered to be the most important measurement property [57]. We could only rate the SDQ and the SDQ-UK on content validity, as some development studies did not involve patients or did not present their results separately for patients with shoulder pain [6, 7, 47, 49, 58, 59]. Originally, the construct of the SDQ was described as “functional status” [5], but the items used were focussed on pain, e.g., “my shoulder hurts when I lie on it,” resulting in a lack of face validity. However, the study which assessed the content validity of the SDQ, used “pain related disability” [43] as the construct to be measured, which would be a more appropriate term. It is therefore important to clearly describe the construct to be measured. All other PROMs did not show much discrepancy between the described construct and its items. However, in case of the SRQ, SDQ-UK and SST, the construct was not described in generally accepted terms (ICF terminology) or an extensive description, which makes it difficult to assess whether the items are an adequate reflection of the construct to be measured.

Most studies focused on validity. However, internal consistency, reliability and responsiveness were also well represented. For hypothesis testing, various comparator instruments were used: shoulder PROMs focused on activity limitation/pain-related disability (e.g., SDQ, SDQ-UK, SRQ, DASH, SPADI), known groups (e.g., medication, specific diagnosis, currently working), general PROMs (e.g., pain intensity, HAQ) and range of motion. An important aspect of the methodological quality assessment is whether the comparator instruments measure the same construct and show adequate measurement properties. We considered that range of motion measures a different construct and we therefore rated studies that solely used range of motion as a comparator instrument as being of poor methodology. We also excluded the comparisons with pain alone and the EQ5D as these also measure a different construct, although in most cases this did not influenced the final ratings.

### Recommendations for future research

Further research is recommended to fill the gaps in knowledge regarding the measurement properties of shoulder-specific PROMs, especially with respect to their content validity, starting with a clear description of the construct, but also whether all items seem to be relevant to patients.

Although all of the evaluated instruments were developed in the 1990s, none of these PROMs showed strong positive evidence for all measurement properties after 20 years of research. Meanwhile, knowledge regarding the development of a PROM has increased and instrument developers must articulate how a particular conceptual framework guided their construct selection, item development (including in-depth interviews and focus groups with patients and experts in the field) and psychometric testing [60]. Also, important issues concerning the limitation of functional activities have changed over time, e.g., computer use is nowadays completely integrated into everyday life, but this is not included in most PROMs. Not only relevant items have been changed, but also the available methodology and technology have reached a new level of sophistication, including “modern” psychometric techniques of item banking, item response theory (IRT) and computer-adaptive testing (CAT) [60]. Recently, the Patient-Reported Outcomes Measurement Information System (PROMIS) was developed using sample qualitative input from patients and IRT methods, to construct and evaluate a preliminary item bank for measuring physical function [61]. At this moment, there are upper-extremity and mobility subdomain scores from the PROMIS physical functioning adult item bank [62].

Computer-adaptive testing has tremendous potential for yielding precise PROM assessment quickly and with significantly reduced respondent burden [60]. The methods of the PROMIS project are likely to substantially improve measures of physical function and to increase the efficiency of their administration using CAT [61].

We therefore propose to develop a new shoulder PROM focused on activity limitations, or evaluate the usefulness of an instrument such as the upper extremity PROMIS scale on patients with shoulder pain, taking new knowledge and techniques into account.

Our study showed that there is a lack of high-quality studies measuring cross-cultural validation. Most often PROMs are being translated, and some measurement properties are assessed. We feel it is of great importance to perform cross-cultural validation for PROMs [57].

## Appendix

(“Shoulder Pain”/OR ((pain* OR complaint* OR disorder* OR lesion* OR injur* OR stiff* OR tight* OR patholog* OR impingem* OR disease*) ADJ3 shoulder*).ab,ti.) OR ((shoulder/OR shoulder joint/OR (shoulder* OR (joint* ADJ3 (glenohumeral OR humeroscapular OR scapulohumeral OR “scapulo humeral”))).ab,ti.) AND (pain/OR “Wounds and Injuries”/OR “Arm Injuries”/OR ((functional ADJ3 (disorder* OR illness* OR impairment* OR limitation* OR disabilit* OR status* OR complaint*)) OR ((activit* OR participat*) ADJ6 (limit* OR complicat* OR interfer*)) OR (Disabilit* ADJ3 Evaluat*)).ab,ti.)) AND (exp questionnaires/OR (questionnaire* OR ((self OR patient*) ADJ3 report*) OR PRO OR PROM).ab,ti.) AND (instrumentation.xs. OR methods.xs. OR validation studies.pt. OR comparative study.pt. OR exp psychometrics/OR exp “outcome assessment (health care)”/OR observer variation/OR exp Health Status Indicators/OR Reproducibility of Results/OR Discriminant Analysis/OR (psychometr* OR clinimetr* OR clinometr* OR (outcome ADJ3 (measure* OR assess*)) OR (observer* ADJ3 variation*) OR reproducib* OR reliab* OR unreliab* OR valid* OR coefficient OR homogen* OR “internal consistency” OR (cronbach* ADJ3 (alpha OR alphas)) OR (item* ADJ3 (correlation* OR selection* OR reduction*)) OR agreement OR precision OR imprecision OR “precise values” OR (test ADJ3 retest) OR (reliab* ADJ3 (test OR retest)) OR stability OR interrater OR intrarater OR ((intra OR inter) ADJ (rater OR tester OR observer OR technician OR examiner OR assay OR individual OR participant)) OR intertester OR intratester OR interobserver OR intraobserver OR intertechnician OR intratechnician OR interexaminer OR intraexaminer OR interassay OR intraassay OR interindividual OR intraindividual OR interparticipant OR intraparticipant OR kappa OR “kappa s” OR kappas OR repeatab* OR ((replicab* OR repeated) ADJ6 (measure OR measures OR findings OR result OR results OR test OR tests)) OR generali* OR concordance OR (intraclass ADJ3 correlation*) OR discriminative OR “known group” OR (factor ADJ (structure* OR analy*)) OR dimension* OR subscale* OR (multitrait AND (scaling ADJ3 analy*)) OR “item discriminant” OR (interscale ADJ correlation*) OR error OR errors OR ((individual OR interval OR rate) ADJ variability) OR (variability ADJ3 (analy* OR values)) OR (uncertainty ADJ3 (measurement OR measuring)) OR “standard error of measurement” OR sensitiv* OR responsive* OR (limit ADJ3 detection) OR “minimal detectable concentration” OR interpretab* OR ((minimal OR minimally OR clinical OR clinically) ADJ3 (important OR significant OR detectable) AND (change OR difference)) OR (small* ADJ3 (real OR detectable) AND (change OR difference)) OR “meaningful change” OR “ceiling effect” OR “floor effect” OR “Item response model” OR IRT OR Rasch OR “Differential item functioning” OR DIF OR “computer adaptive testing” OR “item bank” OR “cross-cultural equivalence”).ab,ti.)
